# Compressive Behavior of Novel Additively Manufactured Ti-6Al-4V Lattice Structures: Experimental and Numerical Studies

**DOI:** 10.3390/ma17153691

**Published:** 2024-07-26

**Authors:** Mohammed Hussein Kadhim Aljaberi, Mohammad M. Aghdam, Taha Goudarzi, Muhannad Al-Waily

**Affiliations:** 1 Department of Mechanical Engineering, Amirkabir University of Technology (Tehran Polytechnic), Hafez Ave., Tehran 15916-34311, Iran; mhaljaberi@aut.ac.ir (M.H.K.A.); tgoudarzi@aut.ac.ir (T.G.); 2 Department of Mechanical Engineering, Faculty of Engineering, University of Kufa, Najaf 540011, Iraq; muhanedl.alwaeli@uokufa.edu.iq

**Keywords:** additive manufacturing, selective laser melting, porous biomaterials, lattice structures, functionally graded

## Abstract

This paper presents novel configurations for additively manufactured lattice structures, including helical and elliptic designs, in addition to the pyramid base model. Functionally graded versions of the pyramid and elliptic lattice structures are developed by considering desirable relative densities in each layer. The lattice structures were manufactured using Ti-6Al-4V powder in a three-dimensional selective laser melting printer. The averaged porosities are 0.86, 0.91, 0.916, 0.93 and 0.74 for pyramid, functionally graded pyramid, elliptic, functionally graded elliptic and helical, respectively. The mechanical behavior of the lattice structures was characterized through compression tests using a universal testing machine and computationally analyzed using finite element code. The results indicate that the elliptic and functionally graded elliptic lattices have elastic moduli of 0.76 and 0.67 GPa, while the yield strengths are 41.32 and 32.24 MPa, respectively, in comparison to cancellous bone. Moreover, pyramid, functionally graded pyramid, and helical lattices show relatively lower elastic moduli of 0.57, 0.65 and 0.41 GPa and higher yield strengths of 54.1, 52.15 and 61.02 MPa, respectively. This could be an indication that they are fit for cortical bones. All samples have low elastic moduli coupled with high yield strengths. This could reduce or eliminate stress shielding, making them suitable for some load-bearing bio-inspired applications. A comparative study utilizing experimental and numerical models was conducted to evaluate the performance of the proposed designs.

## 1. Introduction

Nowadays, people in need of orthopaedic implants are increasing, which encourages industrial engineering researchers to invent and develop orthopaedic devices with optimal compatibility and promising lifetimes [1]. The latest progress in additive manufacturing technology opened the door to a new generation of biomaterials by using the repeated micro-architecture in implants [2]. In the former periods, the implantation of Ti-6Al-4V and Co-Cr-Mo alloys led to the development of an implant that has a predictable lifetime of up to 15 years. However, there are still limitations related to mechanical behavior such as stress shielding, infections, and untying [3]. Various studies have been considered in AM porous biomaterials with the aim of enhancing porous structures by improving pore shape, size, and distribution tissue regeneration performance [4].

Due to the significant role of micro-architecture in controlling the mechanical and biological properties of porous biomaterials, intensive analytical, computational, and experimental studies have been performed to provide a better understanding of their behavior. Porous structures typically involve 3D unit cells, such as tetradecahedron, rhombic dodecahedron [5], and honeycombs [6]. Many types of structures were studied to provide desirable mechanical performance. One of them is lattice-based structures: Ti–6Al–4V cellular structures fabricated using SEBM showed mechanical properties comparable to human bone, minimizing stiffness disparity and potentially reducing stress-shielding effects [7]. Diamond and BCC lattices, particularly those modified by XYZ, displayed superior rigidity and strength, with diamond structures exhibiting properties similar to trabecular bone, making them suitable for porous implants. Varied porosities (60%, 75%, and 90%) in Ti-6Al-4V ELI alloys affected mechanical properties, with diamond and BCC lattices showing favorable stress-compression elongation characteristics [8]. Split-type P lattice structures made from Ti-6Al-4V demonstrated excellent load-bearing capacity and energy absorption, with an elastic modulus ranging from 1.50 to 3.50 GPa and a yield strength ranging from 57.95 to 152.74 MPa [9]. The findings indicate that surface-based topologies are less prone to fatigue failure and may enhance bone growth compared to traditional truss-based designs. Among the tested materials, spinodal surface-based architected materials exhibit the highest performance. These materials can be fabricated through self-assembly followed by material conversion, enabling scalable production of implants with micro-scale unit cells that significantly increase surface area per unit volume and improve bone growth efficiency [10]. A new study demonstrates that in-plane fracture control can be achieved by replacing unit cells in sensitive areas and introduces a novel assembly method for out-of-plane fracture control. This method involves sandwiching different thin plates of metamaterials with varied layouts, promoting multi-step fracture and improving fracture behavior from brittle to ductile. The analysis shows that increased ligament curvature enhances ductility, but excessive curvature can reduce energy absorption capacity [11].

The second type of structure is Triply Periodic Minimal Surface (TPMS) structures. TPMS sheet configurations, particularly polymeric ones, showed enhanced mechanical properties compared to metallic and ceramic-coated microlattices, and were less influenced by density changes [12]. A numerical approach was used to simulate cubic gyroid structures under quasi-static compression, and the influence of geometric factors on structural response was quantified with One Factor At a Time (OFAT) and Taguchi methods. And a gyroid structure was theoretically developed that imitates both elastic modulus and compressive strength of human cortical bone [13]. Three TPMS micro-architectures, D-diamond, G-gyroid, and P-primitive, were produced by 3D printing from hydroxyapatite. The mechanical properties of the specimens were analyzed, followed by their implantation for the study of bone augmentation and osteoconduction capabilities. In terms of strength, the D-diamond and G-gyroid performed significantly better than the P-primitive. As a result, ceramic scaffolds with D-diamond and G-gyroid microarchitectures appear to be the best choice for TPMS-based scaffolds in bone tissue engineering due to the fact that the treatment of bone deficiencies consists of both defect bridging and bone augmentation [14].

The third type of structure is functionally graded structures. Functionally graded BCCz lattices demonstrated superior energy absorption, with 114% additional energy absorbed before full densification compared to non-graded BCCz lattices [15]. Functionally graded double pyramid dodecahedrons exhibited desirable mechanical properties, with the ratio of inclined to uninclined struts affecting elastic modulus and yield stress [16]. Functionally graded porous scaffolds with gyroid and diamond configurations achieved high strength (152.6 and 145.7 MPa, respectively) and an elastic modulus of 3.8 GPa, similar to cortical bone [17]. Multiphasic topology optimization for functionally graded porous structures enhanced structural strength and thermal efficiency, showing promising results in achieving targeted porosity [18].

The effects of geometric structure, cell size, and material type on mechanical properties (modulus and yield strength) are also studied. An inverse relationship between cell size and mechanical properties in rhombic dodecahedron structures was observed, with larger cells resulting in lower elastic modulus and strength [19,20]. Mechanical properties of AM porous biomaterials systematically altered by material type and geometric structure, with Co-Cr structures showing enhanced post-elastic stress levels and energy absorption [21,22]. 

Cellular materials studies have revealed the mechanical characteristics of standard porous biomaterials, which are predominantly influenced by the micro-architecture of lattice structures. Many configurations of unit cells were investigated and modeled, and analytical relations for the mechanical properties were found [23]. 

In conclusion, lattice-based structures, TPMS configurations, and functionally graded designs provide desirable mechanical performance for various applications. Mechanical properties like modulus, yield strength, and energy absorption are significantly influenced by the type of structure, cell size, and porosity. Smaller cell sizes generally lead to higher strength and modulus, while functionally graded designs often enhance mechanical properties such as energy absorption and structural strength. However, their effectiveness depends on the specific application and the precise control of the latest mentioned gradient parameters [24].

In this study, two novel configurations of lattice structures, including helical and elliptic designs, in addition to the pyramid base model are presented, manufactured, and tested. The Functionally Graded (FG) versions of the pyramid and elliptic lattices are also designed by taking into account the desired relative densities within each layer. CAD computer-aided program is used for designing unit cells and lattice configurations. The specimens are manufactured from additive manufacturing powder Ti-6Al-4V using the Selective Laser Melting (SLM) method. Moreover, scanning electron microscopy (SEM) was used to characterize the morphologies of additively manufactured specimens. Mechanical properties of the lattice structures are evaluated by quasi-static compression tests. The computational domains of the lattice and Functionally Graded (FG) lattices are modeled and solved via the multi-physics COMSOL package Ver. 5.5. 

### 1.1. Topology Selections

**Figure 1:**
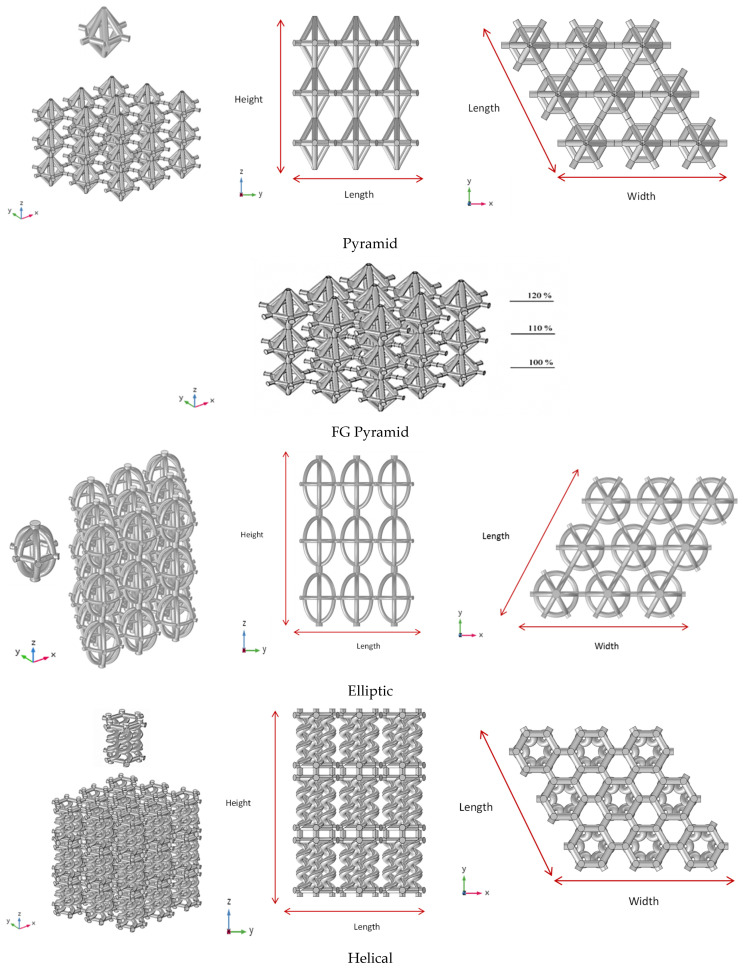
The proposed Ti-6Al-4V three-unit cell configurations.

The basis of the unit cell design is a close representation of the bone in the human femur. The lattice structure morphology is designed based on a single unit cell in a repeating pattern of selected representative volume element (RVE) to mimic the random structure of bone. The proposed unit cells are in three patterns together with two Functionally Graded (FG) versions, as shown in Figure 1. In a standard design, a pyramid unit cell [16,25] is compared with the helical and elliptic novel designs. The helical lattice represents an innovative design concept inspired by the renowned spiral form, while the elliptical shape draws motivation from the symmetrical nature of a biological bird egg. Furthermore, the FG elliptic structure exhibits varying porosity levels across the layers. It should be noted that the elliptic model is modified from the standard pyramid case at the edges of the unit cell in order to minimize the stress concentration, which makes it an appropriate case in real applications.

In the simulation process, appropriate Representative Volume Elements (RVEs) are considered to construct geometry of the lattice structures for each configuration, and the strut diameter is 1 mm for the single unit cell. In addition, the FG versions of the pyramid and elliptic lattice structures with a porosity variant along one direction are designed and fabricated. To create an FG lattice, the diameter of strut cross-sections was changed in each layer. For instance, strut diameters ranging from 1, 1.1, and 1.2 mm for each layer were considered to create functional graded lattices in the pyramid and elliptic cases [16]. As a result, the five studied cases are pyramid, FG pyramid, elliptic, FG elliptic and helical.

### 1.2. Material Selection 

In order to achieve the desired results, a lightweight, strong and biocompatible material was needed. Ti6Al4V titanium alloy, which contains 91% HPC phase and 9% BCC phase, has been chosen for interesting biocompatibility and desirable shear strength. The aluminum and vanadium phases stabilize each other at room temperature [26]. Considering the lower stiffness of the combined bone (1.4 GPa) and the modulus of the Ti6Al4V base material (122 GPa), it is required to develop a structure with a density of 10% and a porosity level of 90%. To produce 1.4 GPa bulk stiffness from a stretch-dominated structure, the overall porosity values must be within this range [27]. 

Once the design parameters were established, each of the three configurations for additively manufactured lattice structures was generated numerically in the X, Y, and Z directions to fill a cubic volume composed of 3 × 3 × 3 unit cells, resulting in an overall height of 10.4 mm for the entire lattice structure. The selection of 3 × 3 × 3 unit cells was based on a previously reported convergence study that ensures minimal edge effects [28]. Another numerical investigation into the suitability of three topologically distinct architected materials for long bone implants also utilized 3 × 3 × 3 unit cells to identify the best mechanical performance [10].

The lattice structure specimens are designed using computer-aided design software (AutoCAD 2020), following which models for additive manufacturing are generated in the (.cad) format. The resulted files are then imported by COMSOL 5.5 for structural analyses.

## 2. Experimental Studies

The specimens are manufactured for each unit cell type, i.e., five lattice structures, using the biomedical Ti-6Al-4V powder. Spherical Ti6Al4V powder, grade 23 according to ASTM F3001 was used [29,30]. Ti-6Al-4V is a widely used material manufactured by the SLM method mainly due to its excellent mechanical properties, low specific weight, and good corrosion resistance. These unique characteristics make it suitable for various applications, such as the medical and aerospace industries. Table 1 illustrates Ti-6Al-4V powder production conditions and mechanical properties [29]. 

### 2.1. Manufacturing and Characterization of Lattice Structures

**Figure 2:**
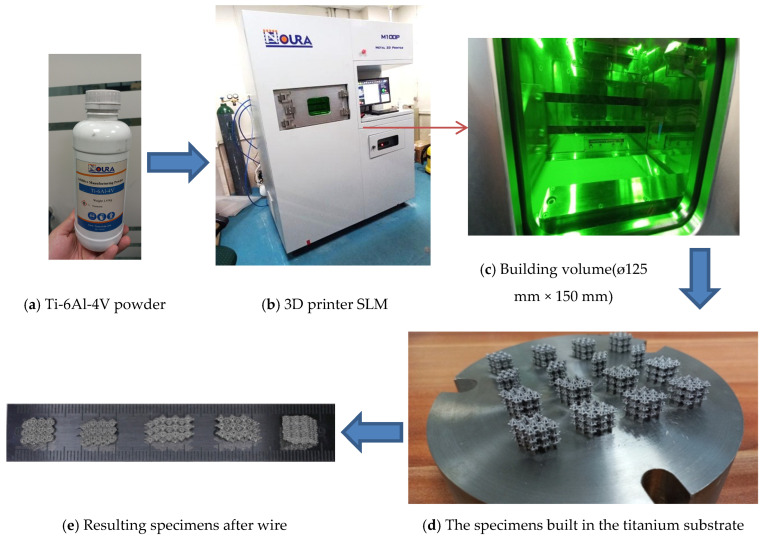

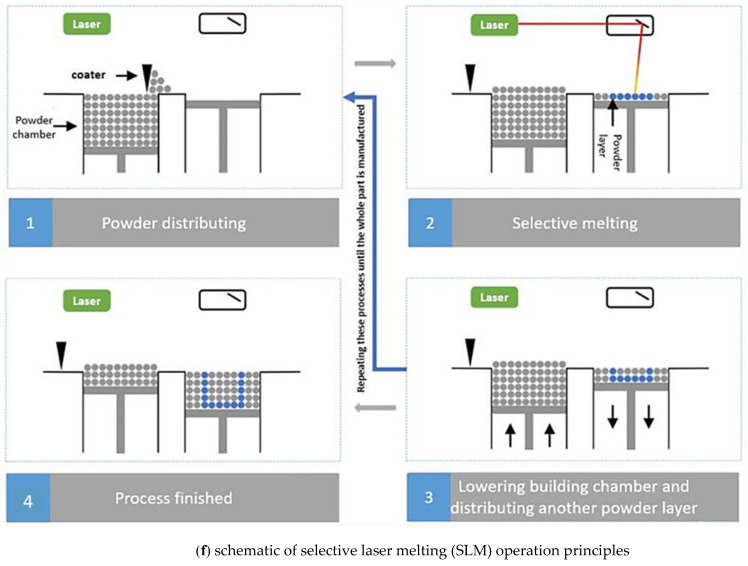
Selective Laser Melting (SLM) technology. (**a**) Additive Manufacturing Ti-6Al-4V powder, (**b**) 3D printer (SLM) machine, (**c**) building volume (ø125 mm × 150 mm) of the 3D printer, (**d**) the specimens built in the titanium substrate, (**e**) resulting specimens attached in the building volume base, (**e**) the specimens after wire separation and (**f**) schematic illustrating selective laser melting (SLM) operation principles [31].

Selective Laser Melting (SLM) is one of the most timesaving prototyping processes for turning ideas into products. Large and innovative companies in the world implement this technology as a flexible, rapid, and cost-effective method to fabricate a product from digital data. The 3D printer that was used to fabricate the specimens was Noura M100P metal with SLM technology, as shown in Figure 2a–f. In Figure 2f, a schematic illustration is presented for operation principles of SLM in which parts are manufactured layer by layer. Various materials including polymers, metal alloys, ceramics, and composite in powder or wire form can be used in this method. Depending on the desired material and geometry, different techniques are implemented to generate adhering layers. One of the most famous techniques uses powder material and laser for the adhering process [31].

**Figure 3:**
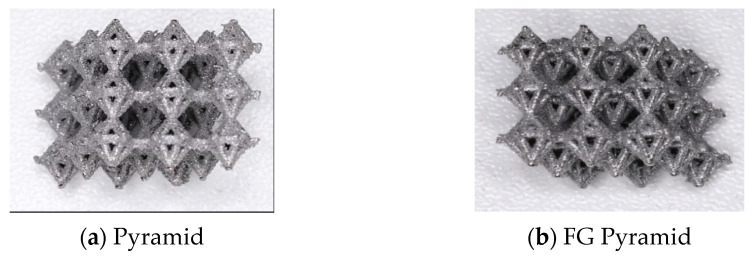

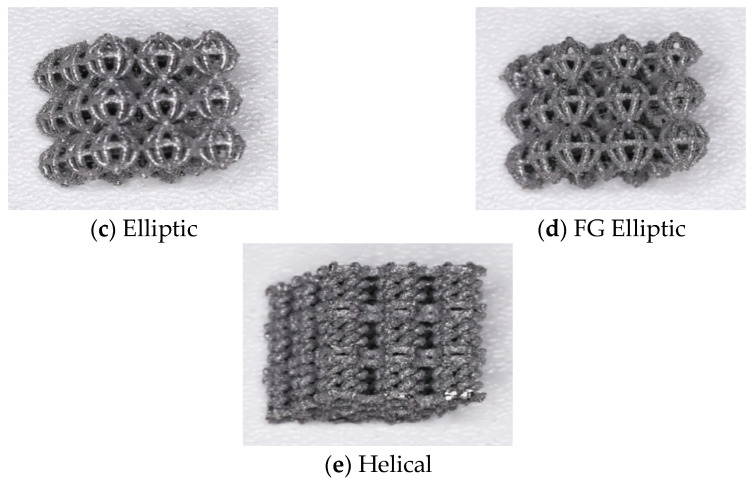
Additively manufactured Ti-6Al-4V specimens.

The production was performed in an inert atmosphere (Argon) and a temperature of 25 °C; each process carried on for 8 h, and samples were built upon a solid titanium substrate. The samples were subsequently removed from the substrate using wire electrodischarge machining. The Ti-6Al-4V specimens from SLM machine are as shown in Figure 2, while their dimensions and porosities are tabulated in Table 2. Resulting specimens are shown in Figure 3. 

With an ø125 mm × 150 mm volume providing this method as a well-matched technique for fabrications of dental, implants industries, and research advancements. Specifications of the SLM machine are [31]:

- Build Volume: Diameter 125 mm, Height: 150 mm
- Laser Specifications: Type: Fiber laser, Power: 300 W
- Optical System: F-theta-lens, scan speed: Up to 7 m/s, focus diameter: 80 µm
- Performance Metrics: Layer Thickness: 20 to 80 µm, Build Rate: Up to 20 cm^3^/h, Dimensional Accuracy: ±0.05 mm
- Environmental Requirements: Inert gas: Nitrogen or Argon, Temperature: 15 °C to 25 °C
- Material Compatibility: Stainless steels, hot work steels, nickel-based alloys, cobalt-chrome alloys, titanium alloys, aluminum alloys

**Figure 4:**
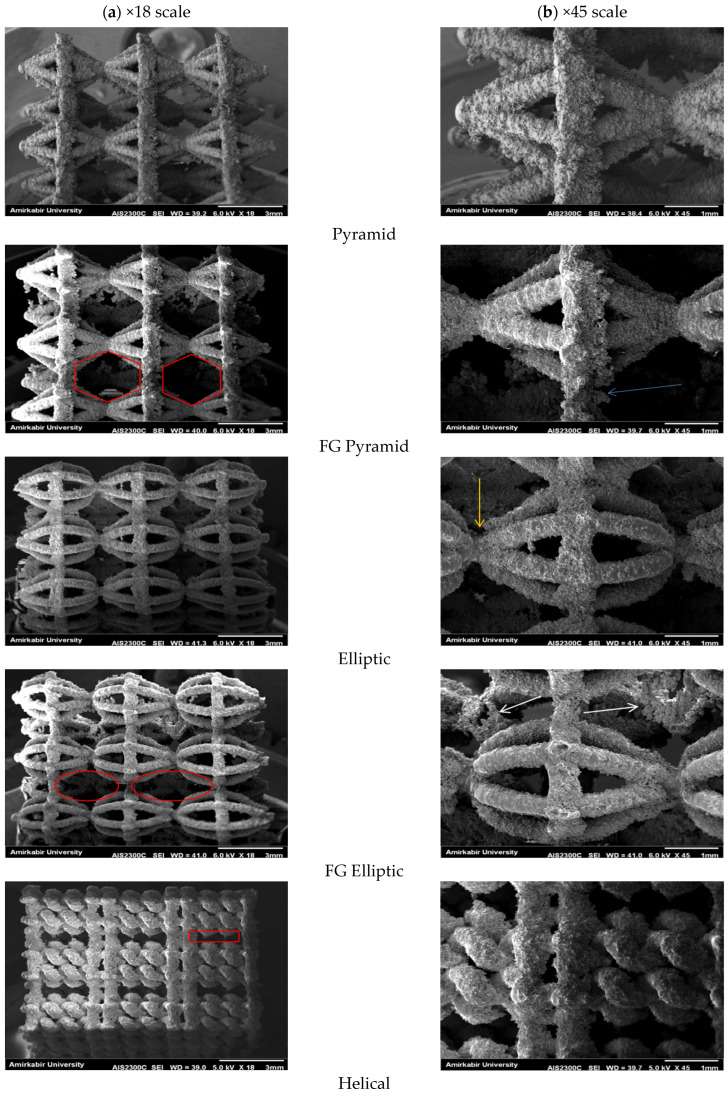
The images of Ti-6Al-4V specimens manufactured using selective laser melting technique: (**a**) ×18 scale and (**b**) ×45 scale.

The Scanning Electron Microscopy (SEM) of SERON technology ALS2100 is utilized to investigate the morphology and defects of the surface. A voltage of 20 kV was used as acceleration voltage, and a frequency of 20.4 Hz was used as collection frequency. The specimens’ topographic characteristics, which were obtained by SEM, are shown in Figure 4. Several strut surfaces showed a distinct partial attachment phenomenon. Important technical specifications of the scanning electron microscopy system are [32]:

- Electron Optics: Electron Source: Tungsten filament or LaB6 crystal, Accelerating Voltage: 0.5 to 30 kV, adjustable, Resolution: 1.5 nm at 15 kV
- Imaging Modes: Secondary Electron Imaging (SEI), Backscattered Electron Imaging (BEI), Energy-Dispersive X-ray Spectroscopy (EDS) for elemental analysis
- Magnification: Range: 20× to 1,000,000×, Continuous zoom capability
- Sample Stage: Type: Motorized 5-axis (X, Y, Z, tilt, rotation), Travel Range: (X/Y: 100 mm, Z: 50 mm, Tilt: −10° to +90°, Rotation: 360°)
- Sample Size: Maximum Sample Size: 100 mm diameter, Maximum Sample Height: 50 mm
- Detectors System: Secondary Electron Detector (SED) for topographical imaging, Backscattered Electron Detector (BSD) for compositional contrast, EDS Detector for elemental analysis
- Performance Metrics

Imaging: High-resolution imaging for surface morphology and microstructural analysis, Depth of Field: High, providing detailed 3D surface topography

Analysis: Qualitative and quantitative elemental analysis using EDS, mapping capabilities for element distribution.

### 2.2. Mechanical Testing 

The compression performance of a metal porous structure is an important mechanical property indicator. Depending on the exercise conditions, the human femur can support 1 to 5 times its weight. Implanted prostheses must possess compressive behavior to satisfy working conditions. Studies observed that in order to meet the relevant mechanical specifications, the porous structures in artificial implant bodies should have a compressive strength of 40 to 50 MPa. 

**Figure 5:**
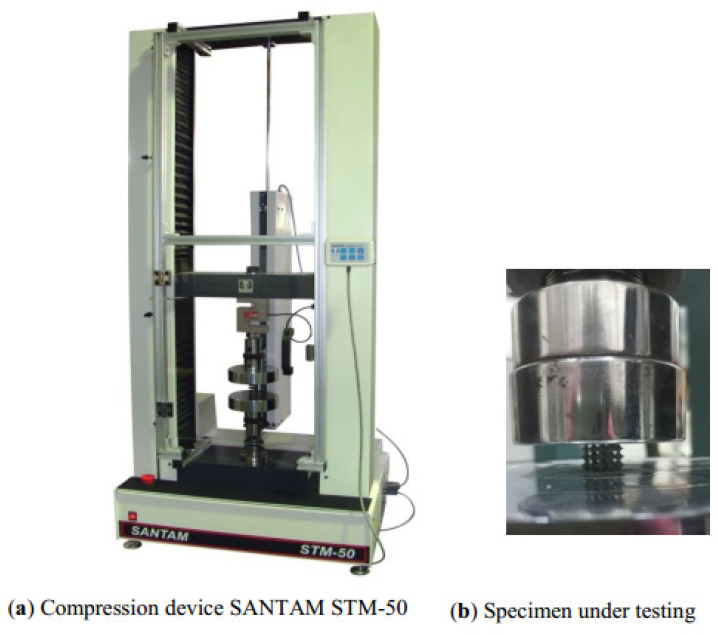
Compression test: (**a**) compression device SANTAM STM-50 and (**b**) specimen under testing.

The mechanical properties are tested and determined by static compression tests via Universal Testing Machine SANTAM STM-50 Servo Electrical Tensile 50 KN compression device, with a 50 kN load cell. The compressive loads were imposed at a rate of 0.5 mm per minute. The specimens are tested in the *Z* direction, to compare the performance of uniform and graded structures. Figure 5 shows both the testing machine and the specimens under compression test. Technical data for the compression test device are as follows [33]:

- Name: Universal Testing Machine -Servo Electrical Tensile 50 KN
- Product Type: Universal Testing Machine
- Product Grouping: Material Science and Engineering Equipment-Material Characterization Analysis and Measurement Equipment-Tensile, Torsion, Bending Test-Universal Tensile Test
- Model: STM-50
- Max. force: 50 kN
- Speed range: 0.001 to 51 mm/min
- Displacement range: 0 to 50 × 0.01 mm
- Piston stroke: 90 mm
- Pressure plate: Ø 250 mm
- Vertical clearance: 325 mm

The slope of the stress–strain graph of specimens in the elastic region is calculated to obtain the elastic modulus. Yield stress can also be estimated by offsetting the line overlaying the elastic part of the stress–strain curve to the right, offsetting by 0.2% strain and finding its intersection with the curve. The area of specimens used for calculations is the cross-sectional area.

## 3. Simulations

**Figure 6:**
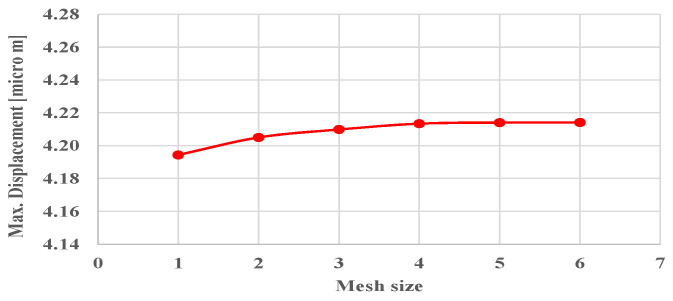
Mesh sensitivity analysis for maximum displacement.

**Figure 7:**
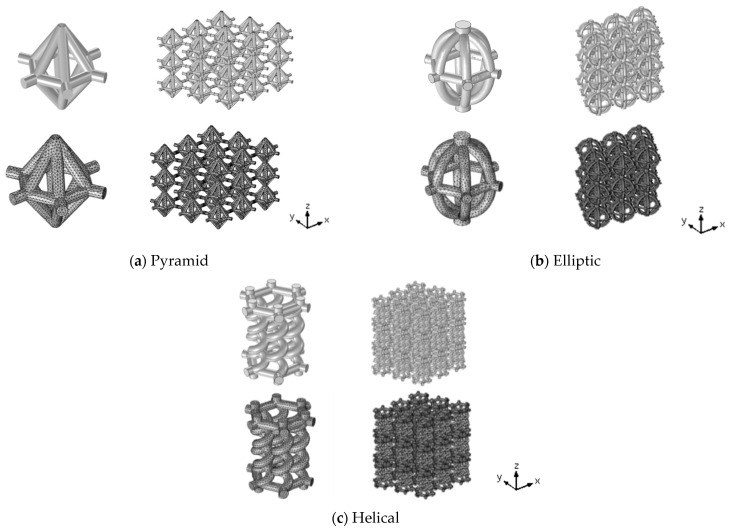
Model and mesh of unit cell and lattice structure for (**a**) pyramid, (**b**) elliptic, and (**c**) helical.

Independency of solutions to the mesh size is studied using a sensitivity analysis for maximum deflection, as shown in Figure 6, and therefore a case of Grid-4 is considered for all analyses. The geometry of the pyramid lattice structure consists of 19,842 domain elements, 7566 boundary elements and 912 edge elements, which show 4.215 µm for max displacement. The proposed configurations of unit cells to construct lattice structures are modeled using quadratic elements for meshing. Figure 7 shows model and mesh for each unit cell and lattice structure of the pyramid, elliptic, and helical configurations, respectively.

Mechanical properties of the bulk material of Ti-6Al-4V used in the numerical study are from [22]: elastic modulus (122 GPa), Poisson’s ratio (0.342), and yield stress (980 MPa) with density of 4430 kg/m^3^.

Boundary conditions are similar to the experimental study: lattice structure bottom nodes are fixed in the *z* direction, while all upper nodes of the structure are exposed to similar displacement under a vertical compressive load of 50 KN. A multiphysics COMSOL mechanical structure code is used to discretize and solve the governing equations. In the finite element analyses, linear elastic with isotropic properties and small deformations are assumed for materials. This numerical model is used to compare experimental results with those predicted by the numerical model.

## 4. Results 

In this section, experimental and numerical results are presented for all types of lattice structures. The load–displacement curves for specimens are obtained from their compression tests. The stress–strain curves are then obtained from load–displacement curves. Mechanical properties such as elastic modulus, yield strength, ultimate stress and maximum displacement results are determined from the stress–strain curve. 

As a result of fitting a straight line with slope in the elastic regime, elastic modulus is derived. The yield strength is determined by finding the point on the stress–strain curve, where it intersects a linear quasi-elastic curve offset by 0.2% strain.

### 4.1. Microscopy

The scanning electron microscopy (SEM) images show the morphology of the surface in the side location of Ti6Al4V structures fabricated by SLM (see Figure 4a,b). The SEM results indicate that the surfaces of all structural types exhibited relatively rough textures due to the adherence of partially melted powder particles. This was caused by non-homogeneous distribution of particle size during the manufacturing process. The precision of the pores and strut size of the FG pyramid and FG elliptic and helical specimens are displayed in the red rings. Images indicated the interconnectivity in the interior of the specimen structures. As an example, the orange arrow characterizes head connection between two adjacent unit cells. Some of the arrows in SEM images indicated by different colors are crack initiation locations with potentially high stress values. For example, the blue arrows characterize side connection between two adjacent unit cells. Additionally, the white arrows characterize dross formation; patches of conglomerated metal can be observed. 

### 4.2. Load–Displacement Validation

**Figure 8:**
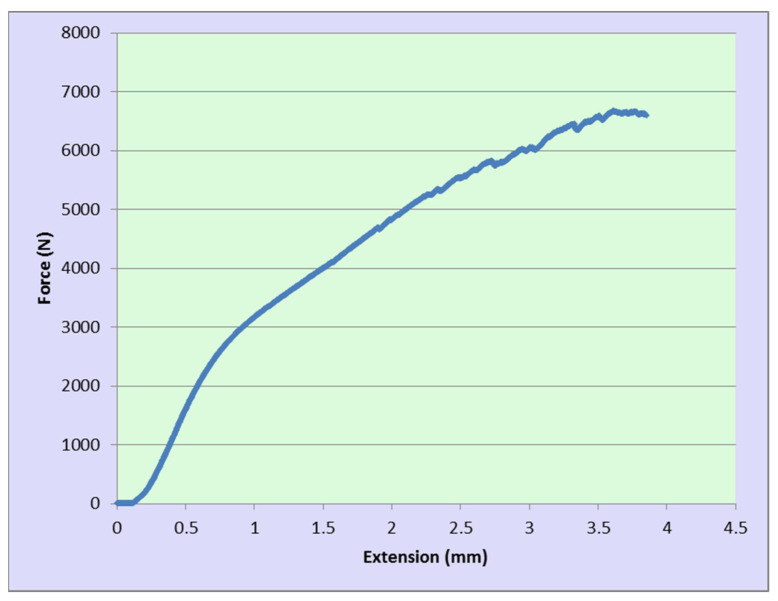
The load–displacement results for pyramid unit cell under compression test.

The load–displacement experiments are utilized to verify the numerical predictions with results collected from the compression test. An example of the experimental outputs for the pyramid unit cell under compression test is illustrated in Figure 8. 

**Figure 9:**
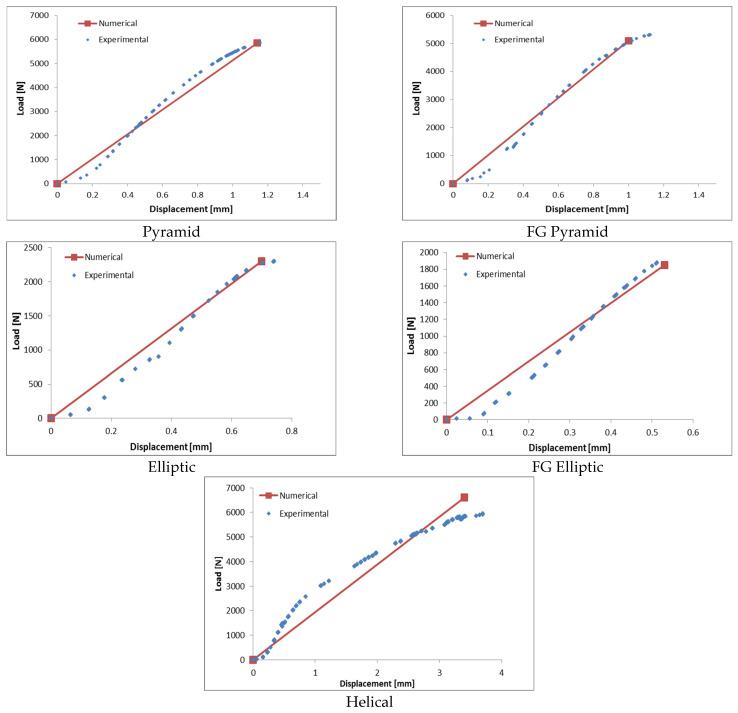
Experimental and numerical load–displacement results for all structures.

Additionally, the load–displacement curves for specimens are acquired through finite element analysis and compared with experimental results, as shown in Figure 9. The findings show that experimental results agree reasonably well with numerical predictions. However, there is a maximum discrepancy of approximately 18% observed for elliptic unit cells at a high porosity of 91.6%.

**Figure 10:**
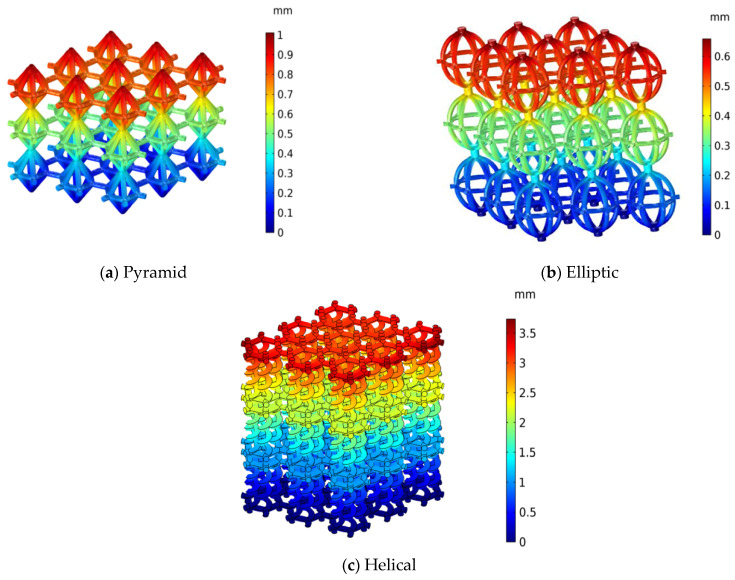
Displacement contours in axial direction for (**a**) pyramid, (**b**) elliptic and (**c**) helical lattices.

Figure 10 presents the contours of displacements in the axial direction at yield point for various lattice structures obtained from the numerical model. The helical lattice exhibited the highest displacement of 3.4 mm at a porosity of 0.74, while the FG elliptic and elliptic designs had the lowest displacements of 0.53 and 0.7 mm, respectively. The pyramid and FG pyramid designs had displacements of 1.14 and 1 mm, respectively. Moreover, all three lattice structures gradually decreased axial displacement starting at the point of load application to the bottom of the fixed surface. Table 3 contains numerical predictions and experimental results for the maximum displacements, which show suitable agreement. 

### 4.3. Stress-Strain Curves

**Figure 11:**
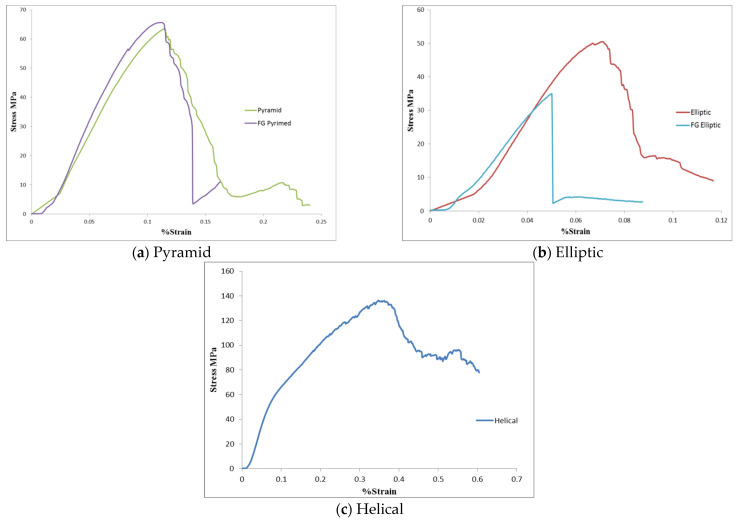
The experimental stress–strain results for lattice structures.

Figure 11 demonstrates the stress–strain curves obtained from the compression test of five specimens. It can be observed that the stress–strain curves of elliptic and FG elliptic specimens show the lowest style, then the pyramid and FG pyramid curves. The stress–strain curve of the helical specimen shows the highest style. The macroscopic corresponding elastic modulus is given by calculating the slope of the linear section of each curve.

**Figure 12:**
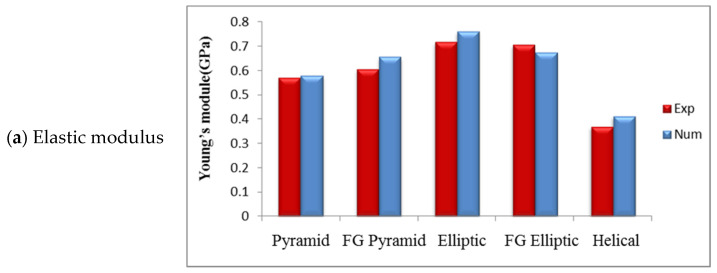

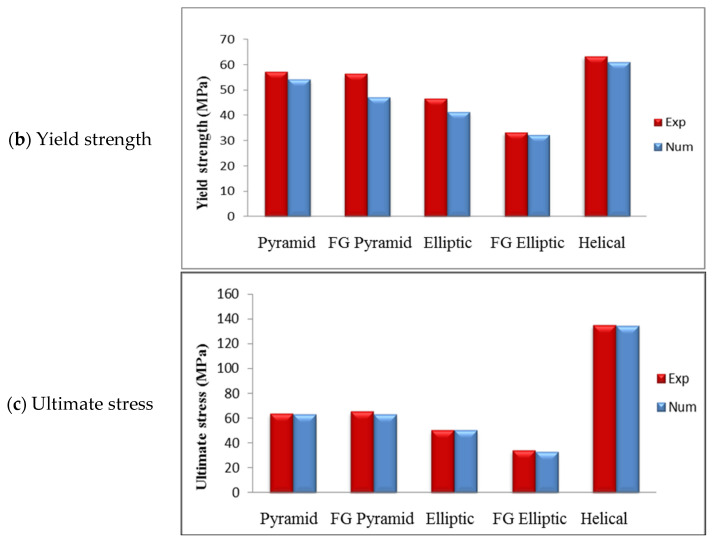
The numerical and experimental results of elastic moduli, yield strength, and ultimate stress for lattice structures.

The elastic moduli of five lattice structures are compared in Table 3 and Figure 12 using experimental compression tests and numerical analyses. According to the results, a good agreement can be found between the values. However, there is a maximum discrepancy of approximately 9.8% observed for the helical elastic modulus results. New proposed lattices with elliptic geometry exhibit the highest elastic moduli, with values of 0.76 and 0.67 GPa for the pure and functionally graded structures, respectively. The pyramid and FG pyramid designs exhibit an elastic moduli of 0.58 and 0.65 Gpa, respectively. The helical design exhibits the lowest elastic modulus of 0.41 Gpa.

Numerical values presented in Table 3 show that the maximum yield stress of 61.02 MPa and ultimate stress of 134.69 MPa occurred in the helical specimen. However, the minimum yield stress of 32.24 MPa and ultimate stress of 33.28 MPa occurred in FG elliptic samples. 

Furthermore, comparison of the numerical and experimental results of yield strength and ultimate stress for lattice structures is presented in Table 3 and Figure 12. It can be concluded that experimental results are in reasonable agreement with numerical predictions. In Table 3 and Figure 12, samples (Exp. And Num.) referred to experimental and numerical results, respectively.

**Figure 13:**
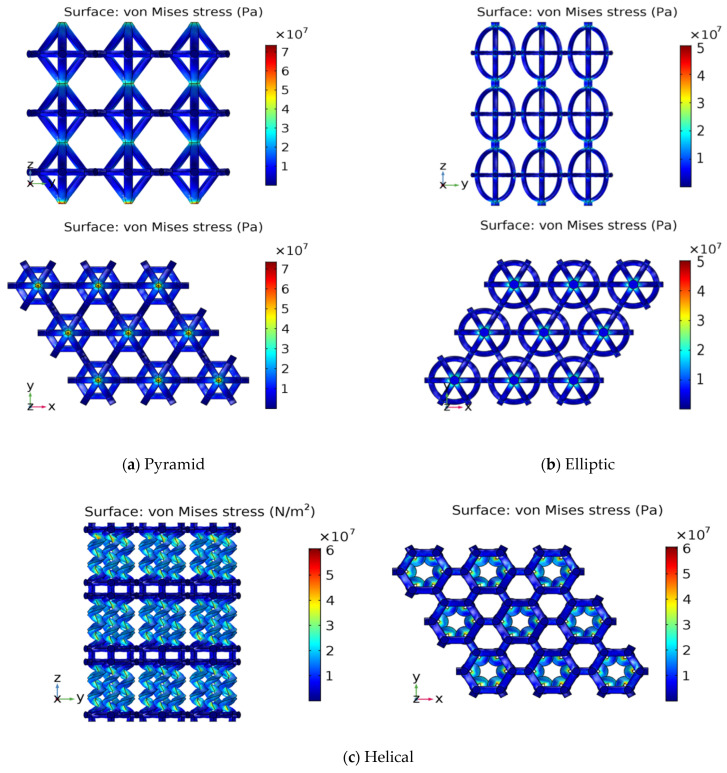
Von-Mises stress distribution in front and top views for (**a**) pyramid, (**b**) elliptic and (**c**) helical lattices.

Figure 13 showed the von-Mises stress distribution in front and top views for the three lattices (pyramid, elliptic and helical). Regions with the highest stress concentration are the likely locations for initial yielding. These regions with the highest von-Mises stress are critical points that need reinforcement or design modification to prevent failure.

## 5. Discussions

As evidenced by the numerical and experimental validation conducted in this study, it was observed that the experimental results exhibited significant deviation from the numerical predictions, as indicated in Figure 9. Surface roughness is a more significant factor contributing to deviations from numerical predictions, especially true when the strut diameter is as small as 1 mm, which is equivalent to the AM process’ resolution limitation. This can be attributed to imperfections in real specimens that are not accounted for in the finite element analyses where idealized specimens are assumed.

The elliptic structure exhibited a higher elastic modulus of 0.76 GPa, which can be attributed to the innovative lattice structure design inspired by the biological egg bird. This design distributes the load on the struts of all three layers of nine unit cells, as depicted in Figure 1. However, despite the higher elastic modulus of the FG elliptic with 93% porosity, the different volumes of unit cells led to the slipping of three layers over each other.

Nevertheless, the minimum displacement observed in the elliptic lattice structure of 0.53 (mm), can be attributed to the novel lattice structure design inspired by the egg bird. The novel helical case has a maximum displacement of 3.4 (mm), which leads to maximum collapse strain, as shown in Table 3 and Figure 10. 

The von-Mises stress distribution plotted in Figure 13 provides a visual representation of stress concentration within a material. By identifying areas with high stress, initial yielding locations can be predicted, and preventive measures can be taken to enhance structural integrity.

### 5.1. Mechanical Properties of Human Bone and Novel Lattice Structures

As reported in the literature, cancellous and cortical bone human own elastic moduli of 0.1–4.5 and 3–20 GPa, respectively. The yield strengths of cancellous and cortical human bones range from 2 to 17 and 33 to 193 MPa, respectively [9]. In the current study, five Ti-6Al-4V lattice structures have mean elastic moduli of 0.41–0.76 GPa, between the values of cancellous bone and cortical bone. The yield strengths of lattices are about 32–61 MPa; these values are identical to cortical bone yield strengths. 

As compared to all five lattices, elliptic and FG elliptic have the greatest elastic moduli of 0.76 and 0.67 GPa and the smallest yield strengths of 41.32 and 32.24 MPa, respectively. These values are within the cancellous bone range for elastic modulus and higher than their reported yield strengths. Moreover, pyramid, FG pyramid and helical lattices have low elastic moduli of 0.58, 0.65 and 0.41 GPa, respectively with high yield strengths of 54.1, 52.15 and 61.02 MPa, respectively. Therefore, the cortical bone would be a suitable choice for these designs. Therefore, the cortical bone is a suitable choice for these designs.

Implant sites must be safeguarded against stress shielding with the use of lower modulus lattices. As bone in-growth takes place, the implant will adjust its mechanical properties by enhancing the structural stiffness and modulus to align with the surrounding bone over time. Biocompatible titanium alloy lattices are ideal for load-bearing applications due to their low elastic moduli and high yield strengths, which eliminate stress shielding [34].

### 5.2. Compressive Behaviour of the FGs

The elastic modulus of the FG pyramid structure (0.65 GPa) is higher in comparison to the pyramid lattice structure (0.58) GPa. Conversely, the elastic modulus value of the FG elliptic structure (0.76 GPa) is marginally lower than that of the elliptic case 0.67 GPa. The predicted and measured elastic moduli of both FG elliptic and elliptic structures are significantly diverse. These deviations may result from variations in strut diameter and porosities from their intended values or because of defects in FG structure manufacturing, as depicted in Figure 12.

The yield strength values of the pyramid and elliptic samples (54.1 and 41.32 MPa, respectively) were found to be higher than their respective FG counterparts (52.15 and 32.24 MPa, respectively), with a noticeable distinction observed in the case of the elliptic and FG elliptic samples. The differences can be attributed to the gradient variation in highly porous structures of FGs, where the diameter of struts increases from approximately 1 to 1.2 mm along the height, leading to a more efficient transfer of compression stress to thicker struts. Notably, the yield strength in FGs is more closely associated with local structural changes than the elastic modulus, as depicted in Figure 12.

This study revealed that both pure and FG samples exhibit the potential to serve as a substitute for natural bone in various locations with varying densities. The incorporation of porosity-graded lattice structures facilitates the fusion of cancellous bone with cortical bone biomaterials. 

### 5.3. Summary of Contribution and Industrial Applications of the Research 

This study offers contributions to the existing materials in the field of AM with the SLM technique and its application in creating advanced lattice structures using Ti-6Al-4V material. For instance, innovative lattice structure designs are introduced and validated, inspired by biological forms, such as the elliptic lattice inspired by bird egg and helical lattice inspired by spiral shape. These designs enhance mechanical properties and distribute loads more efficiently, which is a critical advancement in the development of AM structures. In addition, the new designs were studied by numerical and experimental methods. The research also identifies the significant influence of surface roughness and manufacturing imperfections on the performance of AM structures. Elastic moduli and yield strengths of the specimens are found to be comparable within the range of human cancellous and cortical bone. This finding is important for biomedical applications, particularly in the development of bone implants and prosthetics that can integrate and function seamlessly with human bone.

Furthermore, Functionally graded (FG) of the same lattice structures are manufactured and tested by varying strut diameters and porosities. This gradient approach allows for more efficient stress distribution and better mimics the natural gradation found in human bones.

From an application point of view, we believe that new possibilities are given for biomedical implants and prosthetics with the new designs and their FG counterparts. This could result in reduction of the risk of stress shielding leading to improved integration and longevity of implants. In addition, the presented configurations may be found to be beneficial in industries where lightweight structures are needed. For instance, the aerospace industry would benefit from excellent mechanical properties, low weight, and good corrosion resistance of Ti-6Al-4V. 

In summary, this research makes significant contributions by advancing the design and understanding of lattice structures in AM, particularly using Ti-6Al-4V material. The findings have substantial implications for biomedical and aerospace industries, where improved mechanical properties, customization, and lightweight designs are paramount.

## 6. Conclusions

This study presents innovative lattice structures made of Ti-6Al-4V, including helical, elliptic, and standard pyramid unit cells. Additionally, Functionally Graded (FG) structures were developed by adjusting densities across layers. Five Ti-6Al-4V lattice specimens were additively manufactured using the SLM technique and subjected to compression testing.

The combined experimental and numerical analyses produced promising findings. The elastic moduli of the elliptic and FG elliptic lattice structures were determined to be 0.76 and 0.67 GPa, respectively, with corresponding yield strengths of 41.32 and 32.24 MPa, values that align closely with cancellous bone properties. In contrast, the pyramid, FG pyramid, and helical lattices exhibited slightly lower elastic moduli of 0.58, 0.65, and 0.41 GPa, yet showcased higher yield strengths of 54.1, 52.15, and 61.02 MPa, respectively, rendering them more suitable for cortical bone applications.

Notably, functionally graded pyramids displayed an enhanced elastic modulus compared to standard pyramids, while FG elliptic structures exhibited a marginally lower elastic modulus than their standard elliptic counterparts. Furthermore, the yield strength values of the pyramid and elliptic samples surpassed those of their FG variations, with significant distinctions observed between elliptic and FG elliptic samples. Both pure and functionally graded specimens demonstrate potential for replacing natural cancellous and cortical bones across diverse anatomical sites owing to their low elastic moduli and high yield strengths. This attribute aids in mitigating stress-shielding effects, rendering these structures well suited for load-bearing functions.

It is believed that the idea of developing functionally graded FG lattice structures with novel configurations can be further studied by varying densities of layers to achieve even better mechanical performance and stress distribution. This gradation mimics the natural variation found in bone, providing a more biomimetic approach to implant design. It is also expected to expand the range of mechanical tests to include fatigue, fracture toughness, and long-term durability, which will provide a more complete understanding of the performance of these lattice structures under various conditions.

## Figures and Tables

**Table 1:** Ti-6Al-4V powder production conditions and mechanical properties.

a-Production Conditions	
Parameter	Value
Powder type Ti64-	Gr.23
SLM machine	NOURA M100P
Coater blade type	Soft
Chamber inert gas	Ar
Sieving module	53 µm
Layer thickness	30 µm
Volume building rate	8–13 cm^3^/h
**b-Mechanical properties**	
**Property**	**Value**
Modulus of elasticity	120 GPa
Yield strength	930 MPa
Ultimate tensile strength	970 MPa
Elongation at breakage	16%
Fatigue strength @ 600 MPa	>10,000,000 cycles

**Table 2:** Dimensions, porosity, and for all specimens.

No.	Name	Height (mm)	Length = Width (mm)	Porosity
1	Pyramid	10.4	9.6	0.86
2	FG Pyramid	10.4	9	0.91
3	Elliptic	10.55	6.75	0.916
4	FG Elliptic	10.55	7.4	0.93
5	Helical	10.4	7	0.74

**Table 3:** Comparison of the numerical and experimental results for elastic moduli, yield strength, ultimate stress, and maximum displacement.

No.	Name	Elastic Modules (GPa)		Yield Strength (MPa)		Ultimate Stress (MPa)		Maximum Displacement (mm)	
		Exp.	Num.	Exp.	Num.	Exp.	Num.	Exp.	Num.
1	Pyramid	0.57	0.58	57.2	54.1	63.66	63.47	1.15	1.14
2	FG Pyramid	0.61	0.65	56.5	52.15	65.47	62.96	0.51	0.53
3	Elliptic	0.72	0.76	46.68	41.32	50.46	50.48	0.74	0.7
4	FG Elliptic	0.71	0.67	33.25	32.24	34.28	33.28	1.12	1
5	Helical	0.37	0.41	63.3	61.02	135.11	134.69	3.79	3.4

## Data Availability

The original contributions presented in the study are included in the article, further inquiries can be directed to the corresponding author.
